# IgG4-related autoimmune pancreatitis and sclerosing cholangitis: A case report and literature review

**DOI:** 10.1097/MD.0000000000037922

**Published:** 2024-04-26

**Authors:** Nanping Wang, Peng Zhu, Yue Xiang, Liping Tao, Tao Huang, Zhisong Feng

**Affiliations:** a Department of Gastroenterology, Affiliated Hospital of North Sichuan Medical College, Nanchong City, Sichuan Province, China; b Department of Hepatobiliary Surgery, Fifth People’s Hospital, Nanchong City, Sichuan Province, China; c Department of Pathology, Affiliated Hospital of North Sichuan Medical College, Nanchong City, Sichuan Province, China.

**Keywords:** AIP, diabetes, IgG4, IgG4-related sclerosing cholangitis, liver cirrhosis

## Abstract

**Rationale:**

Immunoglobulin G4-related disease (IgG4-RD) can involve various organs throughout the body, primarily manifesting as endocrine dysfunction, visual impairment, jaundice, and limited sexual function. IgG4-related autoimmune pancreatitis is triggered by autoimmune reactions and characterized by structural changes in the pancreas and pancreatic ducts. The disease mainly affects middle-aged and elderly males, typically presenting as progressive painless jaundice and misdiagnosed as cholangiocarcinoma or pancreatic cancer.

**Patient concerns:**

This study reports a 54-year-old male who consulted with different institutions multiple times due to diabetes, pancreatitis, elevated liver enzymes, and jaundice.

**Diagnoses:**

Magnetic resonance imaging revealed swollen head of the pancreas and atrophic tail. Liver and pancreatic tissue pathology showed IgG4 plasma cell infiltration, while liver biopsy indicated interface hepatitis, liver fibrosis, and pseudolobule formation, with no evidence of bile duct damage.

**Interventions:**

Following hormone therapy, the patient’s serum IgG4 levels and liver enzyme levels returned to normal.

**Outcomes:**

The disease relapsed 2 years after maintaining hormone therapy, and the patient underwent additional hormone-induced remission therapy combined with azathioprine.

**Lessons:**

The purpose of this research report is to enhance the awareness and understanding of IgG4-RD, emphasizing the necessity for personalized treatment strategies that take into account its recurrence, associations, and imaging features. This report provides valuable insights and guidance for clinicians in managing and diagnosing patients with IgG4-RD.

## 1. Introduction

Immunoglobulin G4-related disease (IgG4-RD) is a recently recognized chronic systemic disease that is closely associated with immunoglobulin G4 (IgG4) lymphocytes. There are 4 classes of IgG: IgG1, IgG2, IgG3, and IgG4, with IgG4 levels ranging between 10 μg/mL to 1.4 mg/mL and rarely exceeding 2 mg/mL. Males and older adults generally had higher levels. IgG4 has unique properties among IgG subclasses: it accounts for only 3% to 6% total IgG in the serum of normal subjects, and because of its inability to bind to clq complement, it cannot activate the classical complement pathway, also has low affinity for target antigens.^[1,2]^ IgG4-RD is a complex, immune-mediated, sclerosing inflammatory disease with common pathological, clinical, and serological characteristics. IgG4-RD was first reported in the pancreas and has since emerged in the orbit, salivary glands, lymph nodes, lungs, kidneys, and other organs.^[3]^ A recent case of IgG4-infiltrated hypertrophic meningitis has also been reported.^[4]^ The histopathological features of IgG4-RD include infiltration of IgG4 + plasma cells, storiform fibrosis, and obliterative phlebitis.^[4]^

Although many IgG4-RD patients have multi-organ lesions occurring simultaneously or asynchronously, with different organ-specific pathological characteristics, there are 2 diagnostic criteria for IgG4-RD on which consensus has been reached: (1) serum IgG4 concentrations > 135 mg/dL and (2) infiltration of lgg4 + plasma cells/IgG + cell ratio > 40%, with > 10 IgG4 + plasma cells per high-power field.^[3]^ Most patients with IgG4-related autoimmune pancreatitis (AIP) are diagnosed without biopsy. Due to the lack of biopsy samples, the composite diagnostic criteria for IgG4-RD have relatively low sensitivity for patients with IgG4-related AIP but sufficient sensitivity for IgG4-related Mikulicz disease and kidney disease. Patients who cannot be diagnosed using the composite diagnostic criteria can be diagnosed using organ-specific criteria, indicating that the composite diagnostic criteria and organ-specific criteria are complementary for IgG4-RD.^[3]^ Some IgG4-RD patients may not meet the aforementioned diagnostic criteria for serum IgG4 concentration, and obliterative phlebitis may not be detected in the pathology; however, tissue pathology suggests IgG4 positivity, and hormone therapy is effective.^[4]^

Obtaining pathological tissues is very important in the diagnosis of IgG4-RD, as infiltration of IgG4 plasma cells has been found in some diseases such as rheumatoid synovitis, stomatitis, skin lesions, and cancers with an inflammatory response.^[3]^ Furthermore, high serum IgG4 is expressed not only in IgG4-RD but also in a few other diseases, such as pemphigus vulgaris, bullous pemphigoid, parasitic infections, and atopic dermatitis.^[5]^ An analysis of specimens from 121 patients,^[6]^ including 100 cases of chronic inflammatory diseases (rich in plasma cells) and 21 cases of significant peritumoral/intramural inflammatory reactions, revealed that IgG4-positive plasma cells are a common component of chronic inflammation. The number of IgG4 plasma cells varies widely between different lesions at the same anatomical location and between lesions in different organ systems.

AIP, also known as sclerosing pancreatitis, is pathologically divided into type I and type II.^[2]^Type I refers mainly refer to lymphoplasmacytic sclerosing pancreatitis, and type II is mainly idiopathic duct-centric chronic pancreatitis. In particular, type I is mainly related to IgG4 and often involves the bile duct; much of the research in this field has been conducted by Japanese scholars. For liver damage caused by elevated IgG4, since ANA is mainly of the IgG type, but can also be IgM and IgG A, and even IgG D and IgG E, ANA might be elevated when IgG4 induces liver damage. Whether IgG4-AIH is a subtype of autoimmune hepatitis (AIH) or a manifestation of IgG4-RD liver involvement remains controversial internationally. We found a case of IgG4-RD involving the pancreas, concomitant with hepatitis, cirrhosis, and cholangitis. Simultaneously, we obtained pathological tissues from the liver and pancreas and conducted an analysis and literature review of IgG4-RD cases.

## 2. Case

The patient was a 54-year-old male who sought treatment at the Department of Gastroenterology of North Sichuan Medical College Affiliated Hospital in October 2021 for systemic jaundice, jaundice of the sclera, and generalized itching of the skin. Past medical history: In August 2021, the patient experienced polydipsia and polyuria, which led to the discovery of elevated blood sugar levels at another hospital. The patient was diagnosed with diabetes and received hypoglycemic therapy. In the same month, he visited another hospital because of abdominal pain and elevated serum amylase and lipase levels. He was diagnosed with “diabetes, acute pancreatitis” and received treatment to suppress pancreatic enzymes and control blood sugar. His abdominal pain improved, and he was discharged. However, in October 2021, the patient experienced symptoms such as weight loss, jaundice, yellow eyes, dark urine, and generalized skin itching.

Physical examination revealed that the patient had jaundice of the skin and sclera, with scattered scratching marks throughout the body. Lymphadenopathy was not observed. No edema or mass was observed in either eyelid. The abdomen was soft and flat with no obvious tenderness or rebound pain, and bowel sounds were 3 to 5 times/min.

Laboratory examination: Amylase 18 U/L, lipase 75 U/L, amylase 41 U/L; AST 93 U/L, ALT 257 U/L, indirect bilirubin 41.6 umol/L, ALP 502 U/L, total bile acid 96.9 umol/L, GGT 737 U/L, total bilirubin 174.3 umol/L, direct bilirubin 132.7 umol/L; IgG4: quantitation of immunoglobulin subclasses (IgG4) 14.800 g/L; ESR 63 mm/h, IL-6 6.62 pg/ml, ANA 1:100, negative for the antigen spectrum of autoimmune liver disease (such as anti-mitochondrial antibody-M2), anti-LKM-1, anti-LC-1, anti-SLA/LP, RO-52, nuclear autoantigen SP-100, nuclear pore protein gp210.

**Figure 1.**
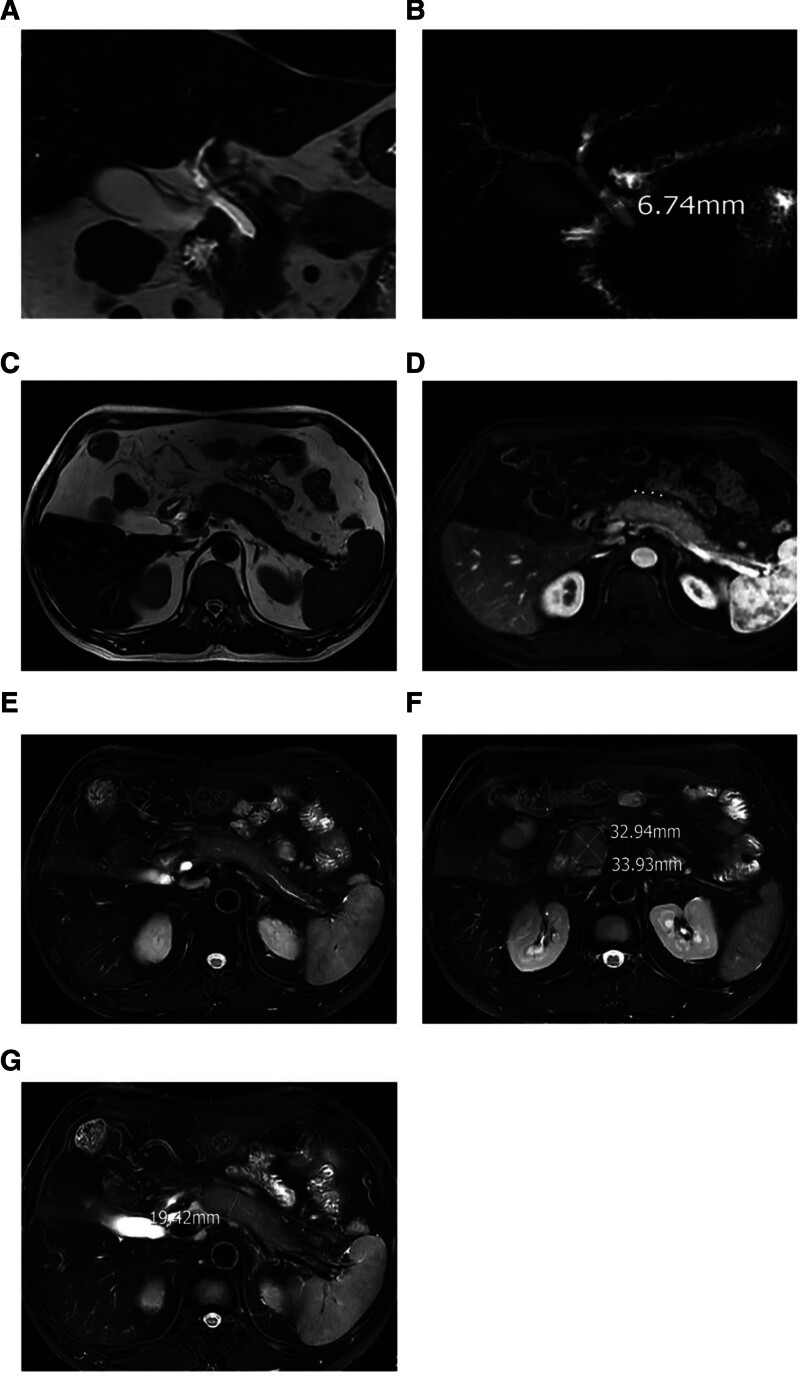
Patient’s upper abdomen MRI + MRCP in September 2021. (A) MRI suggests rat tail-like changes in the common bile duct, narrowing of the downstream of the common bile duct, and upstream dilation. (B) There are segmental narrowings in the hepatic portal region and the intrahepatic left and right bile ducts, narrowing at the lower end of the common bile duct, and the main pancreatic duct is segmentally narrowed with dilation, and the common bile duct in the pancreatic head is narrowed. (C and D) Lesions in the head and body of the pancreas, showing low attenuation soft tissue edges (capsular signs); lesions in the pancreas show soft tissue encapsulation. (E) Swelling of the head and neck of the pancreas, leading to narrowing of the main pancreatic duct in the head and neck, and irregular dilation of the main pancreatic duct in the tail of the pancreas. (F) Enlargement of the uncinate process of the pancreas, with uniform signals. (G) Enlargement of the head and body of the pancreas.

Magnetic resonance cholangiopancreatography showed swelling of the pancreatic uncinate process and body, atrophy of the pancreatic tail, mild dilatation of the main pancreatic duct, and streaky abnormal signal shadows around the pancreas. The pancreatic periphery showed a pseudocapsular sign, with some intrahepatic bile ducts slightly narrowed and the distal common bile duct narrowed, appearing in a rat-tail shape (Fig. 1).

**Figure 2.**
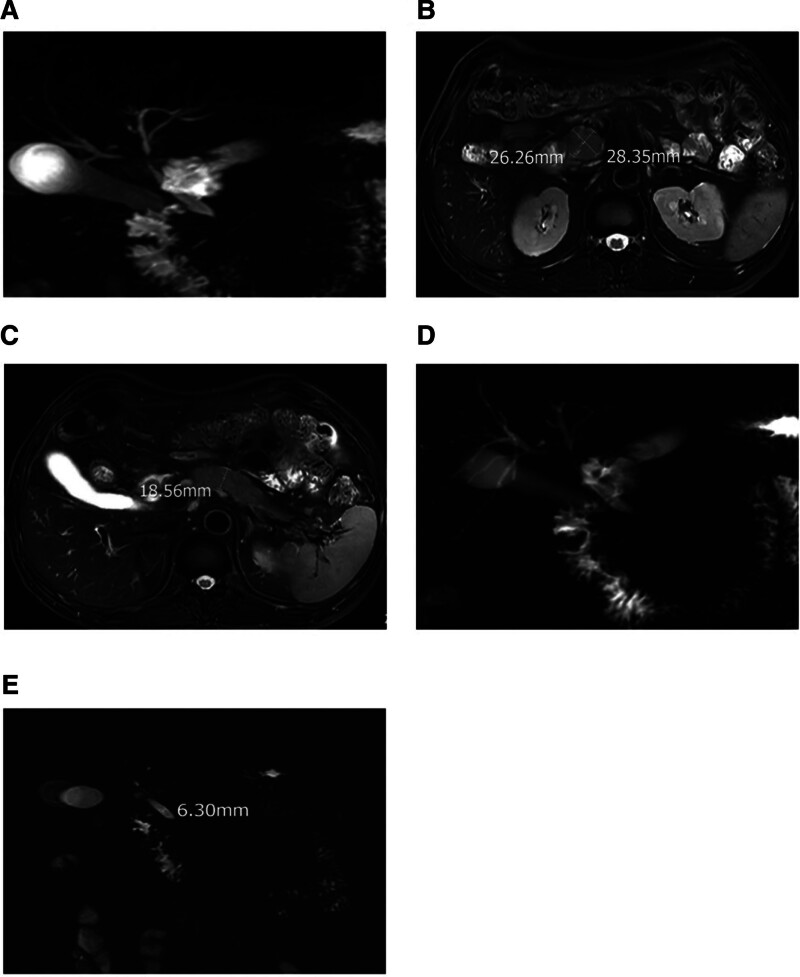
A and B (IHC400×) show liver tissue stained with hematoxylin and eosin (HE) (100×), revealing lymphocyte and eosinophil infiltration in the portal area, as well as bile stasis. C. displays CK7 staining (IHC 200×) in the liver tissue, indicating small bile duct proliferation, suggesting downstream biliary obstruction and bile stasis. D demonstrates liver tissue stained with Masson trichrome (400×), indicating increased collagen fiber formation. E presents reticular staining (200×) in the liver tissue, indicating fibrotic changes with the formation of pseudolobules. F shows IgG4 staining (IHC400×) in the liver tissue. G exhibits pancreatic tissue stained with IgG4 (IHC*200×). IHC = immunohistochemistry staining.

Pathological examination: The patient underwent computed tomography-guided liver biopsy and endoscopic ultrasound-guided fine-needle aspiration (FNA) biopsy of the pancreas. Pathological analysis showed lymphocytes and plasma cells infiltrating the hepatic hilum area, cholestasis, increased collagen tissue, fibrotic changes, and the formation of pseudolobules in some tissues. IgG4 > 10/HPF and IgG4/IgG > 10% were observed in both liver and pancreatic tissues (Fig. 2).

IgG4-related autoimmune pancreatitis (IgG4-AIP) and sclerosing cholangitis (IgG4-SC) were diagnosed based on pathological, laboratory, and imaging findings. The patient was excluded from pulmonary tuberculosis, Epstein-Barr virus, cytomegalovirus, hepatitis B virus, herpes simplex virus infections, and other possibilities. The patient was initially treated with intravenous infusion of methylprednisolone 40 mg/day for 5 days as an induction therapy for remission, followed by oral prednisone 50 mg/day. During treatment, the patient’s bilirubin and obstructive enzymes decreased significantly, but itching persisted, and bile acid levels continued to rise. Therefore, he was treated with Shiwei Dida capsules for choleresis, and his itching symptoms gradually subsided. The steroid dose was gradually reduced to 5 mg and maintained for 2 years.

**Figure 3.**
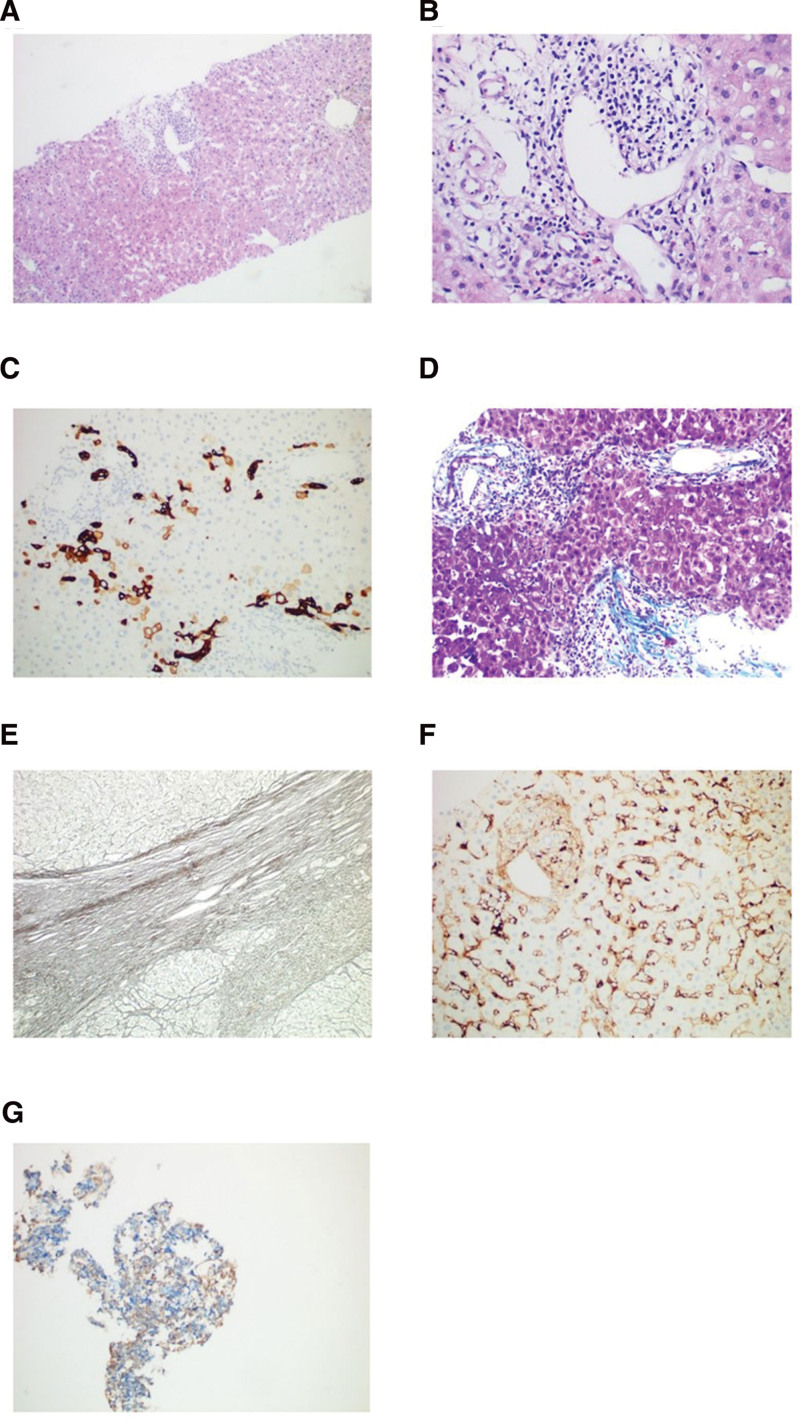
Upper abdomen MRI + MRCP in October 2023. (A) MRI: The uncinate process of the pancreas is still enlarged, but the volume has decreased compared to September 2021 (Fig. 1F). (B) Comparing to the situation of intrahepatic and extrahepatic common bile ducts in September 2021 (Fig. 1B), the narrowing of the hepatic portal bile duct has worsened. (C) The capsular sign of the pancreas is not obvious, and the pancreatic volume has decreased compared to September 2021. (D) The extent of common bile duct dilation has reduced compared to September 2021.

In May 2023, the patient experienced an episode of acute pancreatitis, and serum IgG4 and liver enzyme levels were normal. However, by September 2023, the patient’s condition had relapsed, with dark urine, generalized skin itching, elevated serum IgG4, and elevated liver enzyme levels. A follow-up abdominal magnetic resonance imaging (MRI) showed worsening intra- and extrahepatic bile duct stenosis and a smaller pancreatic uncinate process and pancreas than 2 years ago (Fig. 3). In the current treatment, the steroid was adjusted to methylprednisolone intravenous infusion at a dosage of 80 mg/day for 3 days as induction therapy. Following treatment, the patient’s liver enzyme levels significantly decreased, overall skin itching significantly reduced, and serum IgG4 levels markedly decreased. Subsequently, the treatment was changed to oral prednisone at a dosage of 80 mg/day, along with oral azathioprine at a dosage of 50 mg/bid. After 3 days, the prednisone dosage was reduced to 60 mg/day and continued for 4 weeks, followed by a weekly dose reduction of 5 mg until a maintenance dosage of 5 mg/day was achieved.

## 3. Treatment

IgG4-RD treatment options include systemic corticosteroids, immunosuppressive drugs, biologics, and surgical removal of affected tissues. In addition to contraindications, corticosteroids are recommended as first-line drugs for relief induction in all active or untreated IgG4-RD patients.^[7–9]^ Patients with IgG4-RD are prone to disease recurrence and cancer risk during maintenance treatment. The following is a summary of IgG4-RD related treatment options.

*Corticosteroids*: According to the international consensus on the treatment of autoimmune pancreatitis, the initial dose of prednisolone should be 0.6 to 1.0 mg/kg/day, with a minimum dose of 20 mg/day. Treatment efficacy was assessed for the first time after 2 weeks. Steroid reduction can be considered after 2 to 4 weeks: a decrease of 5 to 10 mg/day every 1 to 2 weeks, then a decrease of 5 mg/day every 2 weeks after 20 mg/day, until the drug is discontinued.^[9]^ Imaging changes in treatment response may appear within 2 weeks.^[10]^ Corticosteroid retreatment is suitable for patients who relapse after successful remission. During maintenance of remission, continuous steroid-sparing therapy should be considered after recurrence.^[8]^ For patients with refractory induction, low-dose hormone pulse therapy (methylprednisolone 500 mg *2, every 4 days, for 2 courses) can be used.^[9]^ However, 31% to 57% of patients experience disease recurrence during steroid tapering or after discontinuation.^[11]^ According to the Japanese AIP consensus guidelines, steroid maintenance treatment is recommended for at least 3 years.^[12]^

*Immunosuppressive agents*: Azathioprine and cyclosporine, can be used as first-line treatment options in addition to corticosteroids. Especially for IgG4-SC, relapses are common after stopping steroids. In a study by Ghazale A,^[13]^ IgG4-SC had a higher recurrence rate for proximal common bile duct lesions, indicating that these patients may require long-term maintenance treatment. Immune modulators, such as azathioprine, can be used to prevent recurrence and maintain remission, and success has been achieved in a few relapsed cases. Azathioprine, mycophenolate mofetil, and cyclophosphamide were effective. Low doses of immunomodulators cannot prevent recurrence in a small number of patients; therefore, higher doses are needed. Azathioprine (recommended–2–2.5 mg/kg or mycophenolate mofetil (750 mg) twice daily.

*The biologics*: CD20 inhibitor rituximab (RTX) reduces B lymphocytes, thereby controlling and improving systemic disease by reducing serum IgG4 + plasma cell levels. Owing to the time it takes for RTX to take effect, jaundiced patients should receive steroid overlap therapy for 4 to 6 weeks. When using these drugs, clinicians should be alert to occult hepatitis B virus or tuberculosis in patients. HBV DNA testing should be performed during drug use.^[9,14]^

*Surgical intervention*: If there is severe biliary obstruction, jaundice, or suppurative cholangitis, percutaneous transhepatic cholangial drainage (PTCD). In some cases, long-term biliary stenting through endoscopic retrograde cholangiopancreatography might be required to reduce jaundice. If pancreatic cancer is present, surgery may be necessary to remove the lesion.

## 4. Discussion

In this case report, the patient sought treatment for diabetes, abdominal pain, and jaundice at multiple hospitals; however, the treatment outcomes were suboptimal and unsatisfactory, with disease recurrence during treatment. Diagnosis of IgG4-RD is extremely challenging. In 1961, Sarles H^[15]^ first described chronic sclerosing pancreatitis with hypergammaglobulinemia. IgG-RD is a new type of IgG4-related disease based on AIP. It is a systemic disease characterized by widespread IgG4-positive plasma cells and T lymphocyte infiltration in various organs. The diseases included in IgG4-RD include AIP, sclerosing cholangitis, cholecystitis, sialadenitis, retroperitoneal fibrosis, interstitial nephritis, interstitial pneumonia, prostatitis, inflammatory pseudotumor, and lymphadenopathy.^[16]^

**Figure 4.**
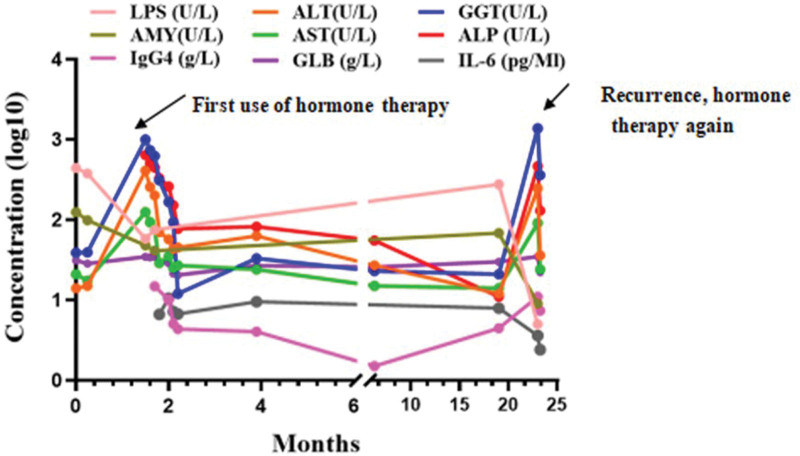
After the use of hormone therapy, the patient’s liver enzymes and serum IgG4 significantly decreased. IL-6 was not significantly related to the remission and recurrence of the disease.

Although the pathogenesis of AIP is unclear, studies have found a significant increase in the number of activated CD4 and CD8 positive T cells carrying HLA-DR in the peripheral blood lymphocytes and pancreatic tissue of AIP patients.^[17]^ Okazaki et al reported that, compared to the control group, CD4 + T cells producing interferon-γ in the peripheral blood of patients with AIP increased significantly and their secretion levels increased. They speculated that AIP may be mediated by the Th1 immune response.^[17,18]^ Kamisawa T^[17]^ pointed out that no AIP antibody has been found so far, and he believed that the pathogenesis of AIP may not involve autoimmunity but may involve other reactions such as allergies. Zen^[19]^ research showed that the expression of Th2 cytokines (IL-4, IL-5, and IL-13) and regulatory cytokines (IL-10 and transforming growth factor-β) was upregulated in the lesion tissues of patients with IgG4-related sclerosing pancreatitis and cholangitis. Therefore, they believed that the allergic mechanism might be the primary response of Th2 and regulatory immune responses in this type of disease.Tsukuda S^[11]^ found that in 43 cases of IgG4-RD, the group with high IL-6 levels was more prone to bile duct damage. They speculated that the level of IL-6 might be related to the patient’s acute inflammatory response level or to concurrent bile duct, liver, and spleen diseases, and might be related to the patient’s prognosis. However, they did not find a correlation between IL-6 level and disease recurrence. Kasashima^[10]^ and others reported that patients with vascular lesions in IgG4-RD more often showed elevated serum IgG4 levels and low fever compared to those without vascular lesions, and they observed a large number of IL-6 positive cells in the tissue samples of affected organs. They speculated that the synthesis of IL-6 might be related to the pathogenesis or progression of IgG4-RD with vascular lesions. Tsukuda S^[11]^ once described a case of AIP with splenomegaly; in this patient, the pancreatic swelling improved significantly after splenectomy. This led them to theorize that the spleen may be involved in the pathogenesis of AIP, as it is an important immune organ. However, in the present case, the patient’s IgG4-related clinical symptoms gradually improved during steroid therapy, and even during the disease relapse phase, we did not find a significant increase in IL-6 levels (Fig. 4). Whether the concentration of IL-6 is truly related to the pathogenesis of IgG4-RD requires further study. In this case, the patient’s jaundice and pruritus improved significantly after receiving steroid therapy, liver enzyme indicators decreased significantly, and serum IgG4 levels decreased significantly. When the disease relapsed, the serum IgG4 levels increased again. This suggests that the concentration of IgG4 may be directly related to disease activity, which is consistent with the findings of Hamano,^[5]^ who found that patients with IgG4-related pancreatitis had high serum IgG4 concentrations and that this value was closely related to disease activity.

The common initial symptoms of IgG4-AIP include painless obstructive jaundice, mild abdominal pain, impaired endocrine and exocrine functions, and recurrent pancreatitis.^[10,20]^ AIP1 is associated with advanced age, male gender, elevated IgG4 levels, involvement of the biliary tract, kidneys, and salivary glands, as well as jaundice as an initial symptom and frequent relapses, according to a multi-center research study investigating AIP.^[21]^ Painless jaundice in AIP patients is due to concurrent bile duct stenosis, which may also lead to the discovery of associated sclerosing cholangitis (IgG4-SC). In addition, various extrapancreatic manifestations may occur, such as salivary gland swelling caused by sclerosing sialadenitis, hydronephrosis, and lymph node lesions caused by retroperitoneal fibrosis.

Radiologically, IgG4-AIP can present in 3 forms: diffuse, multifocal, and focal. Pancreatic diffuse enlargement, glandular fullness, and smooth margins, losing the normal “feather-like” contour, forming a delayed enhancement “sausage-shaped” pancreas, are the most representative manifestations of AIP.^[10]^ Only approximately 30% to 40% of patients can observe low attenuation of peripancreatic soft tissue (capsule sign), which is a very specific manifestation of AIP, usually considered to be the result of inflammatory cell infiltration and fibrosis.^[10]^ AIP may also present as multifocal pancreatic masses, with the pancreatic duct not obviously dilated or diffusely narrowed owing to swelling of the glandular body. In endoscopic retrograde cholangiopancreatography, irregular stenosis of the main pancreatic duct was found.^[20]^ If focal stenosis is found in the pancreatic part of the common bile duct, accompanied by thickening and enhancement, it should be considered whether there is combined IgG4-SC.^[10,22]^

The main tools for diagnosing IgG4-SC are computed tomography, MRI, and EUS, which can be used to detect bile duct wall thickening. EUS and IDUS can provide higher-resolution bile duct wall images. The diagnosis of IgG4-SC also depends on its typical imaging manifestations and pathological tissue biopsy; however, obtaining pathological tissue biopsy samples is often difficult in clinical practice.

Abnormal pancreatic imaging findings, such as peripancreatic capsule sign, atrophy, abnormal enhancement, or T2 signal intensity, strongly favor the diagnosis of IgG4-SC.^[23]^ Khandelwal A^[10]^ states that 90% of AIP cases have concurrent biliary system involvement, with the main pancreatic duct being the most common site of involvement, manifesting as diffuse wall thickening or proximal short-duct dilation. The presence of a penetrating duct sign or icicle sign (pancreatic duct stenosis) further favors the diagnosis of benign stenosis in AIP, although pathological diagnosis is the gold standard.

Cholangiography can reveal stenotic areas. There are 4 types of imaging features of IgG4-SC.^[24–26]^ For the diagnosis of IgG4-SC, there are 4 reference criteria: (1) characteristic biliary imaging findings, (2) elevated serum IgG4 concentrations, (3) coexistence of other IgG4-related diseases besides biliary disease, and (4) characteristic histopathological features. In addition, the effectiveness of steroid therapy is an important criterion for the accurate diagnosis of IgG4-SC. In IgG4-SC, both bile duct involvement and bile duct wall thickening are continuous, in contrast to segmental stenosis characteristic of jumping lesions in PSC.

The diagnosis of IgG4-SC is based on the following 4 criteria^[26]^: (1) characteristic biliary imaging findings, (2) elevated serum IgG4 concentration, (3) coexistence of IgG4-related diseases other than biliary disease, and (4) characteristic histopathological features. An additional diagnostic criterion for the accurate diagnosis of IgG4-SC is the effectiveness of steroid therapy. Continuous bile duct involvement and bile duct wall thickening in IgG4-SC are different from jumping lesions characterized by segmental bile duct involvement in PSC.^[23,27]^

According to a A^[28]^ report by Tanaka IgG4-SC patients, the most common symptom in 527 patients with IgG4-SC is jaundice, followed by itching and abdominal pain. Symptoms of decompensated liver cirrhosis are extremely rare in IgG4-SC patients. Of the 535 patients with IgG4-SC, 485 had serum IgG4 levels, of which 84.3% (409) had serum IgG4 levels above the normal range (<135 mg/dL) and the remaining 15.7% had serum IgG4 levels within the normal range.

The patient presented with painless jaundice accompanied by itching, irregular stenosis of the intrahepatic and extrahepatic bile ducts, significant stenosis at the junction of the left and right hepatic bile ducts, and a rat-tail change in the lower segment of the bile duct. These changes are similar to those observed in the third type of cholangiography mentioned above: perihilar lesions and stenosis of the lower segment of the bile duct.

The characteristics of IgG4-RD involvement in the liver, including IgG4 hepatitis and IgG4-related AIH, remain unclear. IgG4 liver disease is a broad term, including liver lesions associated with IgG4-RD and/or IgG4-SC, primary liver changes inherent in IgG4-RD, liver involvement caused by IgG4-SC, and secondary changes associated with IgG4-SC.^[29]^ There is disagreement over whether IgG4 related liver disease belongs to the subgroup of IgG4-RD or AIH. The development of IgG4-RD may involve the activation of the complement system, leading to a reduction in complement levels.^[30]^ Autoimmune hepatitis related to reduced complement levels of IgG4 has the clinical and histological characteristics of classical autoimmune hepatitis, but its characteristic is a significant increase (>10/HPF) of IgG4 positive plasma cells.^[29]^

The 3 main histopathological features of IgG4-RD include (1) dense lymphoplasmacytic infiltration, (2) focal fibrosis with an onion-skinning appearance, and (3) obliterative phlebitis. Other histopathological features encompass (1) venulitis without luminal occlusion, and (2) an increased number of eosinophils.^[29,30]^ Although immunohistochemistry plays a crucial role in the diagnosis of IgG4-RD, it is essential to interpret the results cautiously because relying solely on the number of IgG4-positive plasma cells can be misleading because of their presence in other inflammatory conditions and even malignant tumors. The incidence of malignant tumors in IgG4-RD patients is relatively high. In a 12-year follow-up study of 158 patients with IgG4-related diseases, 34 developed malignant tumors, with autoimmune pancreatitis being the most common.^[12]^ In this case, CEA and CA125 levels were within the normal range, but mild elevation of CA199 (40.4) was observed during recurrence. Currently, this is attributed to the involvement of bile duct cells causing a mild increase in CA199, and long-term follow-up is needed to determine if there is any malignancy.

It should be noted that IgG4 can cause systemic lesions, and autoimmune pancreatitis is a manifestation of IgG4-related disease in the pancreas.^[20]^ IgG4-RD is often misdiagnosed as a lymphoma, malignant tumor, or other diseases.^[3]^ As IgG4-RD affects various organs, its clinical symptoms differ, and each IgG4-RD patient may present with different clinical symptoms when seeking medical attention to address organ-specific lesions. Therefore, it is particularly important for clinicians to improve their understanding of IgG4-RD. In a study by Asano J,^[12]^ IgG4-RD and AIP were significantly associated with malignant tumors, which often occur within 5 years after the diagnosis of IgG4-RD. IgG4-related autoimmune pancreatitis can lead to an increase in CA199, as IgG4-related diseases can affect multiple organs throughout the body, including the bile duct, liver, and pancreas. Therefore, it is crucial to distinguish it from periampullary cancer. Obtaining a relevant pancreatic histopathological biopsy is particularly important to avoid unnecessary surgery.^[20,21]^ Clinicians must be aware of the diverse manifestations of IgG4-RD as it can affect multiple organs and present with various clinical symptoms that may lead to misdiagnosis. Timely and accurate diagnosis is crucial for appropriate treatment and for avoiding unnecessary surgeries or interventions.

Early stage IgG4-RD can be treated with corticosteroids, whereas late-stage IgG4-SC may not respond to steroid treatment. This is because both AIP and IgG4-SC cases initially exhibit primarily inflammatory characteristics, which then decrease, followed by significant fibrotic scarring in the later stages. Early steroid induction treatment is mainly aimed at relieving symptoms and protecting the function of the related organs. However, secondary atrophy caused by fibrotic changes is irreversible. This should be considered when attempting steroid treatment of IgG4-related diseases.^[10,24,31]^ Regular follow-up imaging and related laboratory indicators are needed during treatment, as many patients have been shown to have concurrent common bile duct cancer, pancreatitis, portal hypertension, liver cirrhosis, liver failure, or even liver transplantation during treatment. In a multicenter study on autoimmune pancreatitis (AIP), it is recommended to consider testing fecal elastase-1 to evaluate pancreatic exocrine function during the treatment process. This study also found that the use of immunosuppressants and biologics in treatment cases was relatively low. The treatment approach described in the study, which used immunosuppressants, can serve as a reference for other doctors treating similar cases.^[21]^

IgG4-RD can involve multiple systemic diseases including vision, secretory glands, thyroid, gonads, lymph nodes, liver, biliary tract, pancreas, brain tissue, and retroperitoneal fibrosis. In patients with multiple system involvement, routine exclusion of connective tissue and hematological diseases is necessary. To diagnose IgG4-RD, clinical manifestations should be combined with serum tests, imaging, pathology, immunohistochemistry, and cytokine examinations. In addition to the primary manifestations of target organ involvement, whole-body screenings for lymph nodes, skull MRI, salivary glands, thyroid, and the endocrine system should be performed. Multi-center, prospective, and retrospective studies are beneficial for better decision-making and treatment strategies in clinical practice, ultimately benefiting patients.

## Author contributions

**Data curation:** Yue Xiang.

**Methodology:** Liping Tao, Tao Huang.

**Software:** Zhisong Feng.

**Writing – original draft:** Nanping Wang, Peng Zhu.

**Writing – review & editing:** Nanping Wang, Peng Zhu.
